# Preference-based crisis care is an infrastructure problem: lessons from joint crisis plans and advance choice documents for Japan

**DOI:** 10.1192/bjo.2026.12059

**Published:** 2026-07-24

**Authors:** Teruyuki Nomura, Shunsuke Kanou

**Affiliations:** Department of Psychological Science, Faculty of Psychology and Welfare, Niigata University of Health and Welfare, Niigata, Japan; Department of Social Welfare, Faculty of Comprehensive Welfare, Tohoku Fukushi University, Sendai, Japan

**Keywords:** Advance choice documents, joint crisis plans, shared decision-making, implementation science, coercion

## Abstract

Crisis planning based on stated treatment preferences should not be judged by trials that never verified delivery infrastructure. Lessons from UK joint crisis plans and advance choice documents (ACDs) suggest outcomes depend on facilitation, accessibility, specificity and exception governance. Japan should test ACDs as measurable crisis-care infrastructure, not paperwork.

**Figure:**
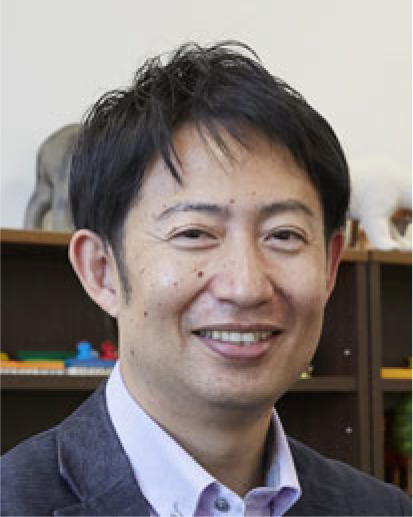

## Crisis preferences are an infrastructure problem

Mental health crises compress time, communication and choice. Under these conditions, preferences are easily lost, and care shifts towards risk management and coercion. Preference-based crisis planning refers to the process of eliciting, recording and acting on a person’s treatment and care preferences for future crises. It includes tools such as joint crisis plans (JCPs), advance choice documents (ACDs) and psychiatric advance directives. The problem is not simply whether individuals have written preferences. It is whether services can create, access, interpret and act on these preferences when crises occur.

Japan, with its high psychiatric bed capacity^1^ and policy shift towards shorter stays, stronger safeguards and community-based care, illustrates this challenge. The Act on Mental Health and Welfare for Persons with Mental Disorders – Japan’s principal mental health law – governs voluntary and involuntary psychiatric care and has historically framed involuntary admission in terms of medical care and protection. Its 2022 amendment, with key provisions implemented in April 2024,^2^ marks a shift towards stronger safeguarding of rights, including measures to prevent abuse in psychiatric hospitals and a novel inpatient visitation support service intended to function as advocacy. However, admission and treatment pathways remain documentation dependent, and documents are not always accessible during crises. The challenge is design. How are preferences created, stored, accessed and acted upon within real workflows?

## Why the JCP record does not settle the question

Evidence for preference-based crisis planning is often inconclusive. A 2022 Cochrane review of shared decision-making interventions found the certainty of evidence to be low or very low across most outcomes,^3^ although it did not include JCPs. A randomised trial of both patient-advocate and clinician-facilitated crisis plans found no effect on admissions or emergency visits,^4^ and a systematic review of crisis-planning interventions reached similarly mixed conclusions across studies.^5^ These results have been read as evidence that preference-based tools do not work. However, we argue that this interpretation is premature.

The trials share a common design problem: they evaluated tools whose implementation conditions – facilitation, specificity, accessibility and exception governance – were not systematically ensured. Judging whether preference-based crisis planning works based on trials in which the delivery infrastructure has never been verified is analogous to evaluating a medication whose administration is left to chance. Null or mixed findings cannot clearly distinguish tool failure from implementation failure when the key delivery conditions are not verified. The question is not whether these tools work but whether they have been tested under conditions that would allow them to work.

JCPs were developed to structure shared decision-making by clarifying service user preferences in advance.^6^ Early trials suggested that JCPs could reduce compulsory treatment,^6^ but the larger CRIMSON trial did not replicate this, though the therapeutic relationship improved modestly.^7^ Preference-based tools can fail quietly: plans may not be updated, may be too vague to guide action,^8^ may not be retrievable in emergencies or may be overridden without transparency. Psychiatric advance directives were accessed in only approximately 20% of crisis events, with a 67% concordance rate when accessed.^9^ Similar problems have been reported in emergency medicine.^10^ These findings reinforce that preference-based crisis planning is a design problem, not a documentation problem.

## ACDs as supported crisis-care infrastructure

ACDs are structured records of a person’s preferences regarding future mental healthcare intended to guide decisions when capacity or communication is impaired. The value of ACDs lies not in adding another form, but in reframing crisis preferences as a supported and accountable process: drafting, updating, sharing across teams, governing exceptions and reviewing use after a crisis. The key shift is from documentation to infrastructure.

In England, recent mental health law reforms have made this shift more explicit. Section 130M of the Mental Health Act 2025 sets out duties for NHS England and integrated care boards to support people in making ACDs,^11^ treating implementation as part of the legal architecture rather than leaving it as optional paperwork. Scotland and the Australian state of Victoria provide useful comparators; their advance-statement frameworks require preferences to be considered and include mechanisms for supporting access to statements and recording or explaining departures from them.^12,13^

Across evidence from JCPs, shared decision-making theory and implementation science,^14–16^ four design requirements recur (Table 1): facilitation capacity, requiring trained facilitators with protected time; actionable specificity, so that preferences can guide clinicians who may not know the person across emergency departments, general hospitals and out-of-area wards; transparent governance of exceptions, reflecting principles of reciprocity and accountability by specifying who may override preferences, under what conditions, with recorded rationale and timely explanations to the person; and accessibility embedded in routine workflow because if an ACD is not accessible during a crisis, it does not exist in functional terms. Accessibility requires redundancy rather than a single repository; clinician-held records support institutional access, whereas patient-held portable copies can bridge service boundaries. Table 1 lists process indicators for assessing whether each requirement is met in a pilot.

**Table 1:** Core implementation requirements and process indicators for preference-based crisis planning, illustrated through a proposed pilot in Japan Table 1 long description.

Design requirement	Operationalisation in a pilot	Process indicators
Facilitation	Named facilitator (e.g., care coordinator or designated community psychiatric nurse) assigned at outpatient follow-up, with protected time (e.g., 60–90 min for first drafting and 30–45 min for updates). Annual scheduled review and event-triggered review after ED attendance, admission or major treatment changes. Monthly supervision and a standard workflow (draft → team sign-off → share → review). Escalation protocol: ED/inpatient staff must check the record, contact the facilitator (or on-call proxy) and document whether the plan was used.	Completion rate among target group, facilitator assignment recorded, time from eligibility to first completed plan, proportion receiving scheduled review and proportion receiving event-triggered review.
Specificity that travels	Structured template with crisis triggers, early signs, de-escalation strategies, preferred and non-preferred interventions and ‘next-best’ options; portable one-page summary for ED/general hospitals; clinician-facing wording for unfamiliar teams.	Crisis-time access recorded, concordance rate between recorded preferences and delivered care when feasible, proportion with ‘next-best’ options completed and clinician-rated actionability/completeness.
Exception governance	Explicit criteria for override, named decision authority, mandatory documentation of rationale, timely explanation to the person and post-crisis review to amend the plan.	Override events with rationale recorded, proportion with documented explanation within time frame, post-crisis review completion rate and amendment rate after crisis.
Accessibility embedded in routine workflow	Single agreed access route with clear flags in records, role-based access controls, portable version held by the person and routine confirmation during stable periods.	‘Plan checked’ recorded at key entry points, successful access rate in emergencies, update within 12 months or after major transitions and portable copy issued and verified.

ED, emergency department.

## What Japan should test now

Japan should not adopt ACDs as fixed paperwork. These should be tested as measurable implementations in defined local systems. Recent policy changes provide practical hooks: the emerging model of a designated community psychiatrist (*kakaritsuke seishinka i*) explicitly positions routine confirmation of a crisis plan in everyday care as part of preparedness for acute exacerbations and emergencies.^17^ Three constraints must be addressed from the start.

First, Japan’s healthcare system offers multiple entry points for crisis care, including emergency departments, general hospitals and psychiatric services, making the responsibility for initiating, locating and activating an ACD unclear. Pilots must define the target populations and explicit activation roles at key entry points. Second, service provision is fragmented across organisations and settings. A staged approach is realistic: begin with a portable version plus local protocols and then expand cross-organisation access only where governance and consent pathways are workable. Third, the evidence base is still under development – an opportunity to treat implementation conditions as testable hypotheses and build measurements into pilots from day one.

A practical next step is a staged pilot in a defined region or provider network, packaging essential components: named facilitation with protected time, reliable access routes across settings, explicit rules for exceptions and routine post-crisis reviews. Early evaluation should prioritise whether the system functions in crisis pathways, rather than relying solely on distal outcomes. A minimal indicator set could include ACD completion among the target group, documented crisis-time access, a recorded override rationale and post-crisis review completion.

These constraints are not unique to Japan. They characterise many high-income health systems in which preference-based crisis planning remains aspirational rather than operational. The design requirements proposed herein – facilitation, actionable specificity, governance of exceptions and accessibility – are intended as a transferable framework. Japan’s proposed pilot strategy offers a tractable model for any system seeking to move from policy intent to measurable implementation.

Preference-based crisis care should not be considered a document intervention. Inconclusive trial records reflect untested infrastructure and not futile tools. The central question is whether these services can deliver conditions that allow preferences to shape care during crises. For Japan, the next step is the staged, measurable testing of ACDs as crisis-care infrastructure aligned with ongoing reforms. The lessons are not only for Japan; any system that takes crisis preferences seriously must build the infrastructure to carry them.

## Table 1 Long description

A table with three columns and five rows detailing core implementation requirements and process indicators for preference-based crisis planning. The columns are labeled ‘Design requirement’, ‘Operationalisation in a pilot’, and ‘Process indicators’. The rows are labeled ‘Facilitation’, ‘Specificity that travels’, ‘Exception governance’, and ‘Accessibility embedded in routine workflow’. Each row provides specific details under each column header. For example, under ‘Facilitation’, the ‘Operationalisation in a pilot’ column mentions named facilitator, annual scheduled review, and escalation protocol. The ‘Process indicators’ column lists completion rate, facilitator assignment recorded, and time from eligibility to first completed plan. Similar detailed information is provided for the other rows.

Navigate back to Table 1.
